# A rare presentation of multiple myeloma with concurrent paraskeletal extramedullary thoracic plasmacytoma: A case report

**DOI:** 10.1016/j.lrr.2025.100501

**Published:** 2025-02-13

**Authors:** Saja I. AbuGhannam, Celina R. Andonie, Yousef Abu Asbeh, Aliaa’ Khalili

**Affiliations:** a Faculty of Medicine, Al Quds University, Jerusalem, Palestine; b Thoracic Surgery Department, Al-Ahli Hospital, Hebron, Palestine; c Hemato-Oncology Department, Dura Hospital, Hebron, Palestine

**Keywords:** Multiple myeloma (MM), paraskeletal plasmacytoma (PP), extramedullary plasmacytomas (EMP), extramedullary disease (EMD), chest wall mass

## Abstract

Multiple myeloma is a hematological malignancy that results from the proliferation of abnormal plasma cells, typically invading the bone marrow but occasionally involving other areas of the body. We present a rare case of a 58-year-old male patient who presented with right-sided thoracic wall mass, which was eventually diagnosed through imaging and biopsy as paraskeletal extramedullary plasmacytoma with concurrent multiple myeloma. The patient exhibited symptoms of chest pain and swelling, with radiological features of a large right-sided chest wall mass. The diagnosis shows the fact that radiological presentations are quite nonspecific, often mimicking other malignancies. Such cases, therefore, require further assistance from thoracic surgery and interventional radiology in addition to advanced imaging techniques such as FDG- PET. He was subsequently treated with a three-drug regimen (VTD-Zometa protocol) including velcade, thalidomide, and dexamethasone, considering radiation versus complete surgical exicion due to the size of the mass. This case also supports the idea that clinical diversity exists among multiple myeloma and that considering paraskeletal extramedullary plasmacytoma must be given due importance when dealing with differential diagnosis of chest wall tumors in old age, although extremely rare. Early diagnosis and exclusion of all other possible diagnoses are also critical for the best possible treatment planning and outcome.

## Clinical practice points

• Paraskeletal extramedullary plasmacytoma can co-occur with multiple myeloma, presenting as a chest wall mass, a rare clinical manifestation.
• Paraskeletal extramedullary plasmacytoma could present with localized chest pain and swelling, as seen in this case.
• Paraskeletal extramedullary plasmacytoma should be considered in the differential diagnosis of chest wall masses in older patients.
• Treatment with the VTD-Zometa regimen (bortezomib, thalidomide, dexamethasone) is effective; surgery or radiation may be considered for large masses.

## 1 Introduction

Multiple myeloma (MM) is a cancerous condition characterized by the proliferation of clonal plasma cells in the bone marrow. This results in a number of clinical characteristics, including osteolytic lesions, infiltration of the bone marrow, abnormal production of proteins, and immune deficiency. As a result, the tumor, its byproducts, and the body's response will eventually result in various organ dysfunctions and symptoms, such as bone pain or fractures, renal failure, increased susceptibility to infection, anemia, hypercalcemia, and, less frequently, clotting abnormalities, neurological manifestations, and features of hyperviscosity [1].

However, MM may affect other parts of the body as well. Chest involvement is frequently seen and may take various forms: skeletal abnormalities, including osteolytic, osteoporotic, and pathological fractures; intra- and extramedullary plasmacytomas (EMP); pulmonary infiltrates, including infections of the lungs; and pleural effusions [2].

Plasmacytomas can be categorized as osseous (medullary) or non-osseous (extramedullary). EMP are rare, make up less than 5% of all plasma cell tumors, and may present as primary lesions or be associated with multiple myeloma [3]. Extramedullary thoracic plasmacytoma is especially challenging to diagnose. Radiological features of this tumor are nonspecific, with CT or MRI appearances closely resembling those of primary or metastatic carcinoma, sarcoma, lymphoma, and neuroendocrine or neuroectodermic tumors [4].

We describe a case of a patient who presented with symptomatic thoracic wall mass, which was diagnosed as EMP with concurrent multiple myeloma. This case is presented due to the rarity and to highlight the varied presentation of MM.

## 2 Case report

A 58-year-old male non-smoker presented with a complaint of right-sided chest pain and discomfort that progressively worsened over three months, accompanied by generalized weakness. A few weeks later, he developed significant swelling and tenderness on the right side of his chest. The swelling developed gradually and slowly increased in size over time. The pain was constant, moderate in intensity, and localized primarily to the upper part of the right chest wall without radiation. There was no history of fever, weight loss, cough, shortness of breath, or hemoptysis. Gastrointestinal symptoms, including dysphagia, odynophagia, hematemesis, or changes in bowel habits, were also absent.

On examination, the patient was hemodynamically stable, with an oxygen saturation of 96% on room air and a heart rate of 78 beats per minute. Inspection of the chest revealed asymmetry of the chest wall, with significant swelling over the right anterior chest. There were no changes in the overlying skin color, no visible dilated veins, ulceration, or discharge. Upon palpation, the mass was smooth, firm, non-tender, lacked fluctuation, had irregular margins, and was fixed to the underlying structure with a normal temperature of the overlying skin. Chest auscultation revealed diminished breath sounds on the right side of the chest. Laboratory investigations showed the following results: hemoglobin 15.2 g/dL, white blood cell count 7.7 × 10³/μL, platelet count 159 × 10³/μL, erythrocyte sedimentation rate 20 mm/hr, blood urea nitrogen 15 mg/dL, serum creatinine 0.94 mg/dL, serum lactate dehydrogenase 285 IU/L, and serum calcium 9.3 mg/dl. The serum total protein level was 8.3 g/dL, with serum kappa and lambda light chain levels of 421 mg/dL and 225.8 mg/dL, respectively, and FLC ratio of 1.86. Immunoglobulin A was 237 mg/dL, immunoglobulin G was 1923 mg/dL (slightly elevated), and immunoglobulin M was 59 mg/dL. The patient had chronic active hepatitis B, with a positive hepatitis B surface antigen (HBsAg 189 × 107), and was on tenofovir therapy. The anti-hepatitis C virus antibody and the antibody against human immunodeficiency virus were non-reactive.

Urinalysis and microscopic urine examinations were normal.

**Fig. 1:**
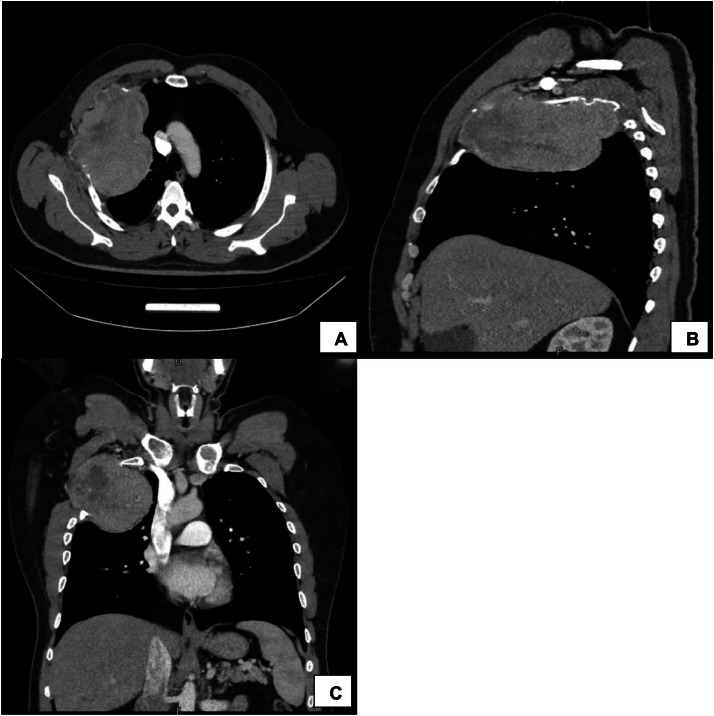
A: Axial B: Sagittal C: Coronal CT scan with contrast of the thorax showing a homogeneously enhancing soft tissue mass measuring 14 × 11 cm in the right upper anterior chest wall with pathological destruction of the right second rib.

The chest radiograph showed an expansile growth arising from the right chest wall (Fig. 1). As part of the workup, the patient underwent pulmonary function testing, with the following results: forced expiratory volume-1 117%, forced vital capacity 116%, and transfer factor for carbon monoxide 166%. Fluoro-deoxy-glucose positron emission tomography (FDG-PET) scan with oral contrast demonstrated a hypermetabolic mass on the right chest wall, causing a bulge in the underlying pleura without evidence of pulmonary parenchymal invasion. No other areas of pathological uptake were noted.

**Fig. 2:**
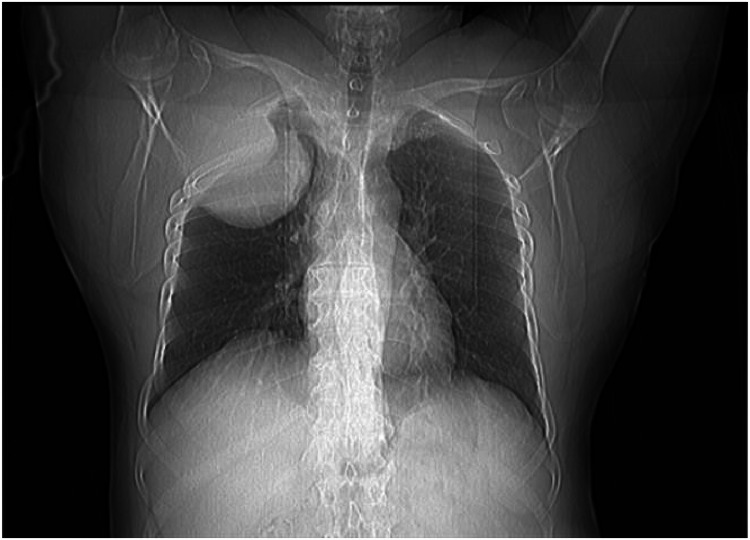
Chest radiograph showing homogenous opacity involving the right upper zone.

A chest CT scan with IV contrast revealed a large right-sided chest wall mass with both intra- and extrathoracic extension, primarily involving the right second rib. The mass appeared to be destroyed and also involved the first and third ribs, as well as the adjacent chest wall muscles. It measured approximately 14 × 11 cm in maximal axial dimensions and showed heterogeneous contrast enhancement. The mediastinum was not affected (Fig. 2). The radiologic findings were highly suggestive of chondrosarcoma, and a biopsy was performed to obtain a definitive diagnosis. The pathology report revealed the presence of neoplastic plasma cells, which were positive for CD138 and lambda immunostains. A bone marrow biopsy demonstrated features consistent with plasma cell myeloma, restricted to the Kappa light chain. CD138 highlighted aggregates of myeloma cells, comprising 15–20% of marrow cellularity, which were positive for Kappa and negative for Lambda light chain. Thus, the diagnosis of our patient was MM showing dual expression of kappa and lambda light chains, with kappa expression in the bone marrow and lambda expression in the plasmacytomas. This is a highly unusual presentation, as only six cases have been reported in the literature [5]. The patient was started on a three-drug combination regimen of Velcade (bortezomib), Revlimid (lenalidomide), thalidomide was given instead of lenalidomide due to the shortage of medications, and dexamethasone (VRd). He showed clinical response since the first cycle.

## 3 Discussion

EMP is the monoclonal growth of plasma cells in soft tissues or organs [6]. It contributes to 3–5% of plasma cell neoplasms, with 80% affecting the upper respiratory tract [7]. They can be solitary or co-existent with MM at the time of diagnosis in 7% of cases. In an additional 6% of cases, it can develop during the disease. EMP has significantly shorter progression-free and overall survival rates [8]. Although plasmacytosis is not uncommon in the bodies of patients who have died of MM, the dissemination of this disease beyond the bone marrow is exceedingly rare [9]. Extramedullary disease (EMD) in MM can arise either from hematogenous dissemination of clonal plasma cells or directly from bone lesions. When tumor cells spread hematogenously, plasmacytomas can develop in soft tissues or organs without involving the bones [10], mostly affecting the skin, subcutaneous tissue, breast, pleura, liver, kidney, lymph nodes, and central nervous system [11]. In contrast, disruption of cortical bone can lead to PP, which forms in the soft tissues surrounding skeletal lesions [10], commonly affecting the ribs, sternum, vertebrae, pelvis, and skull [11]. Rarely, plasmacytomas can develop from areas of previous traumatic injury, such as surgical scars or bone fractures. The incidence at diagnosis was 17.6% for PPs and 1.9% for EMPs, with a recent increase in both due to improvements in the sensitivity of imaging techniques [12]. The most common clinical manifestations encountered by patients with extramedullary chest wall plasmacytomas are chest pain and localized palpable swellings in the chest wall, which is consistent with our case [8].

Although EMD plasmacytoma presents a diagnostic challenge, particularly if the thoracic vertebrae or ribs are not involved, diagnostic imaging is essential in its diagnosis [6]. Magnetic resonance imaging (MRI) is useful for determining the extent of soft-tissue involvement and differentiating PPs from EMD [13]. The radiologic appearance of EMD can be nonspecific, with CT or MRI features comparable to those of other malignancies like primary or metastatic carcinoma, sarcoma, lymphoma, and neuroendocrine tumors [6]. Furthermore, FDG-PET is the most effective imaging modality in MM, with a special focus on detecting PP and EMD. Whole-body PET/CT is still the modality of choice regarding imaging [14], followed by an incisional biopsy of the mass to confirm the diagnosis of plasmacytoma [4,8].

According to the NCCN 2024 guidelines, solitary plasmacytomas (PPs) are treated with either surgical excision or irradiation. However, if PPs are associated with other lesions or co- present with multiple myeloma (MM), they are best treated with the VRd regimen or a combination of daratumumab, bortezomib, melphalan, and prednisone (VMP), rather than proceeding immediately with autologous stem cell transplantation (ASCT) [11,15].

## 4 Conclusion

Extramedullary plasmacytoma (EMP) should be considered in the differential diagnosis of chest wall masses in older patients, despite its rarity and the limited number of reported cases in the literature. Early detection and differentiation from other potential diagnoses are essential, as they have significant implications for prognosis and treatment planning.

## Ethical approval

Not Required.

## Informed consent

Gained from the patient.

## Disclosure

The authors reported no conflict of interest.

## CRediT authorship contribution statement

**Saja I. AbuGhannam:** Conceptualization, Visualization, Writing – original draft, Writing – review & editing. **Celina R. Andonie:** Conceptualization, Visualization, Writing – original draft, Writing – review & editing. **Yousef Abu Asbeh:** Conceptualization, Supervision, Writing – review & editing. **Aliaa’ Khalili:** Conceptualization, Supervision, Writing – review & editing.

## Declaration of competing interest

The authors declare that they have no known competing financial interests or personal relationships that could have appeared to influence the work reported in this paper.
